# Accident Cases

**Published:** 1908-06-06

**Authors:** 


### Resident Medical Officers' Department.

### ACCIDENT CASES.

The occurrence at the Northampton General Hospital, a short *résumé* of which appeared in The Hospital of January 25, affords a suitable opportunity for a brief consideration regarding resident medical officers and accident cases.

Before proceeding to a general consideration of the question perhaps the record of a case which recently came under my notice may first be given. A man was brought to a hospital with the history that he had fallen off a dray, one of the wheels of which was alleged to have passed over his abdomen. He was conveyed to hospital on the dray. With the assistance of a companion he walked without much difficulty from the outside into the casualty room. On examination very little could be made out. The man was quite conscious, and he was able to answer when questioned, although he showed a disinclination to speak, and volunteered no information unasked. He was perfectly sober. There was a small contused superficial wound at the back of the head. There was no bleeding from the ears, nose or mouth, and the pupils were equal and reacted. There were no signs or symptoms of fractured skull or severe injury of the brain.* On inspection of the abdomen there was an indication that a wheel had passed over it. No pain was complained of, palpation was not resented, and percussion failed to reveal anything abnormal. Examination for fractured pelvis was negative. The net result of the examination was that the man had this small wound on the back of his head, was suffering from a certain amount of shock, and, though not unconscious, he took little interest in what was going on. I hesitated about admitting the case, but as I had one or two empty beds at my disposal I thought it better to err on the side of safety, and I therefore admitted him. Half an hour later, when he had been put to bed, I again examined him, but could not make out anything definite. Instructions were given that he should be kept quiet and carefully watched. In about an hour the sister of the ward reported that he was worse. In the interval he had passed urine without difficulty, and there was no blood in it. He now complained of severe general abdominal pain. Palpation was painful, not specially so in any particular region. He had severe tenesmus, and he asked for the bed-pan three times. Once he passed some clotted blood when straining over the bed-pan. He still was able to answer when spoken to, but his general condition was much worse. His pulse was very small, rapid and irregular, his respiration sighing, he was sweating profusely, and his extremities were cold. I was just about to get the things to transfuse him when the man died, less than two hours after admission. In reporting the matter to the coroner I stated that I thought there were internal injuries, but that I was unable to state what they were. The coroner did not, however, order a post-mortem, and I could not get the consent of the relatives to make an examination.

This case illustrates well how difficult it sometimes is to determine whether or not an accident case should be admitted. There was no indication when the man was first seen that there were injuries of so serious a nature. Had this case been sent home there would have been a great fuss, and probably I should have been censured by the jury. I afterwards found out from the relatives that constitutionally the man was a wreck. He had been abroad for some years, and when there he had had malaria; and he had more or less severe relapses at intervals ever since. He was a confirmed drunkard; indeed, only two months previously he had been under medical treatment for an attack of delirium tremens. In a person whose constitution had been thus undermined it is within the bounds of possibility that shock alone might account for death, but from the symptoms which supervened before death it seems probable that there was internal hæmorrhage, possibly from rupture of one or more of the abdominal viscera. One is inclined to ask, "Is it possible for the wheel of a cart to pass over the abdomen without leaving some external mark?" I think it is possible, for about eighteen months ago, when resident in a children's hospital, I met with a case which appears to support this statement. A boy was brought to the hospital with the statement that the wheel of a cart had passed over the lower part of his abdomen. There was no external evidence of this on the abdomen, but he had pain on pressure over the hypogastric and iliac regions. There was no evidence of fractured pelvis, but the child was admitted for observation (I may say that his clothes bore testimony to the fact that the wheel must have passed over him). On the following day the whole of the lower part of the abdominal wall showed a mass of bruised tissue. Apart from this no other injuries were detected, and the child made a quick and an uninterrupted recovery.

Patients who have received severe injuries will often refuse to stay in hospital, and in such cases it is well to get them to sign what I call a responsibility form. This is a declaration couched in language such as this: "I hereby declare that I have been advised to come into hospital, but I have refused, and I accept all responsibility for so doing." Incidentally I may mention that if any in-patients insist on leaving against the advice of those in charge, I invariably make them sign a somewhat similar declaration. By doing so one not only protects oneself, but also the hospital. In the case of children the person responsible for the foolish act should sign the declaration.

Occasionally one sees in medical journals annotations under the title "drunk or dying"—such paragraphs often being called forth owing to a fatal ter-

* One cannot always judge the nature of head injuries from the patient's general appearance, as is well demonstrated by the following case: A man working on the railway was struck on the head by a rod on an engine and rendered unconscious, in which state he remained for several hours. On the following day he was sent to hospital. On admission he was quite conscious. On examination there was a small scalp wound, and on probing this a depressed fracture could be detected over the motor area. The only symptom that the patient had was *partial* paralysis of the left leg, and yet when the wound was opened up there was found a large piece of bone driven on to the brain. When the depressed and splintered portions of bone had been removed there was an opening in the vault $3\frac{1}{2}$ by $1\frac{1}{2}$ inches.

mination having occurred in a case which was diagnosed as drunk, but in which there was serious injury or disease. These cases are very difficult ones for newly qualified men to distinguish between, and in any doubtful cases it would be well to admit them for observation, as by so doing the regrettable mistake of refusing admission to a person with a fracture of the base of the skull or some such serious injury would be avoided. Some " chiefs " fail to recognise how difficult it is for a recent graduate to diagnose at once between coma due to alcohol and coma due to injury to the brain; and when a student I have heard surgeons on more than one occasion make fun of their residents for admitting cases unnecessarily. It may afford the " chief " an opportunity for displaying his power of sarcasm, but it is not very pleasant for the " R.M.O." to hear the students addressed in the following way: " Gentlemen, this case came up late yesterday evening, and our friend, Mr. So-and-So, has been good enough to give the patient a night's lodging."

When seeing round a hospital in the Midlands recently I noticed an arrangement that might well be adopted in every hospital. There were two small rooms with a bed in each, and the casualty officer could detain any doubtful cases in these rooms for the night. If the coma was due simply to alcohol the patient was allowed to go home next morning without being entered as an admission; while if there were injuries of a serious nature, then the case was sent to the ward of the surgeon whose receiving week it was. If this plan were universally adopted then these occasional regrettable occurrences would be practically unknown.

